# MicroRNAs Regulating Oxidative Stress in Human Fertility: A Narrative Review of Mechanistic Insights and Clinical Potential

**DOI:** 10.3390/medsci13040254

**Published:** 2025-10-31

**Authors:** Ioanna Vassilaki, Anastasios Potiris, Ekaterini Domali, Theodoros Karampitsakos, Despoina Mavrogianni, Themos Grigoriadis, Athanasios Zikopoulos, Efthalia Moustakli, Argyro Papadopoulou, Ismini Anagnostaki, Eriketi Kokkosi, Nikolaos Thomakos, Sofoklis Stavros

**Affiliations:** 1 Medical School, National and Kapodistrian University of Athens, 11527 Athens, Greece; jeannettevas@windowslive.com (I.V.); isanagnostaki3@gmail.com (I.A.); 2 Third Department of Obstetrics and Gynecology, University General Hospital “ATTIKON”, Medical School, National and Kapodistrian University of Athens, 12462 Athens, Greece; theokarampitsakos@hotmail.com (T.K.); sfstavrou@med.uoa.gr (S.S.); 3 First Department of Obstetrics and Gynecology, Alexandra Hospital, Medical School, National and Kapodistrian University of Athens, 11528 Athens, Greece; kdomali@yahoo.fr (E.D.); dmavrogianni@med.uoa.gr (D.M.); tgregos@med.uoa.gr (T.G.); argypapadopoulou@gmail.com (A.P.); nthomakos@med.uoa.gr (N.T.); 4 Torbay and South Devon NHS Foundation Trust, Lowes Bridge, Torquay TQ2 7AA, UK; thanzik92@gmail.com; 5 Department of Nursing, School of Health Sciences, University of Ioannina, 45500 Ioannina, Greece; ef.moustakli@uoi.gr; 6 Department of Midwifery, Faculty of Health and Caring Sciences, University of West Attica, 12243 Athens, Greece; ekokkosi@uniwa.gr

**Keywords:** infertility, oxidative stress, miRNAs, redox equilibrium, biomarkers

## Abstract

Human infertility represents a multifaceted condition, with oxidative stress (OS) and microRNAs (miRNAs) emerging as key contributors to its pathophysiology. This comprehensive review explores the complex interplay between reactive oxygen species (ROS) and miRNAs in male and female reproductive dysfunctions. ROS overproduction damages DNA, lipids, and proteins, impairing sperm quality and oocyte maturation. In males, OS is a leading cause of infertility, especially in conditions like varicocele, where key miRNAs such as miR-21, miR-34a, and miR-155 are dysregulated. In females, oxidative imbalance affects granulosa cells and follicular environments in disorders such as PCOS, premature ovarian insufficiency (POI), and endometriosis. Several miRNAs (e.g., miR-132-3p, let-7, miR-642a-5p) regulate mitochondrial function, steroidogenesis, and apoptosis through redox-sensitive signaling pathways (PI3K/Akt, NF-κB, FOXO1). Their altered expression in ovarian and seminal environments correlates with poor reproductive outcomes. Emerging evidence supports their potential role as diagnostic biomarkers and therapeutic targets, although most findings are based on animal models or in vitro studies. This review highlights the therapeutic potential of miRNA modulation and calls for further clinical research to validate miRNA-based interventions. Ultimately, understanding the miRNA–OS nexus offers promising avenues for improving diagnosis, prognosis, and treatment of infertility across both sexes.

## 1. Introduction

According to international data infertility affects 10–15% of couples who attempt to conceive for twelve consecutive months of unprotected intercourse. The two primary factors in infertility consist of equal contributions from male and female components, while oxidative stress (OS) and epigenetic microRNA regulation represent fundamental elements. The microRNA molecule functions as a post-transcriptional regulator of gene expression and plays essential roles in cell functions such as proliferation, differentiation, apoptosis, and stress response [1]. This paper presents a narrative review that integrates mechanistic and clinical findings on the interplay between microRNAs and OS in infertility, synthesizing data across human, animal, and in vitro models.

OS occurs when ROS accumulate and disrupt redox homeostasis, which subsequently damages DNA, proteins, and lipids, leading to impaired reproductive cell function [2]. This review investigates the molecular interaction between miRNAs and its effect on human fertility. Oxidative stress causes significant damage to reproductive health by generating excessive ROS and impaired antioxidant defense systems. Male infertility represents a significant worldwide public health concern since it causes 40–50% of all infertility cases in couples [3]. OS constitutes the leading cause of male infertility because it develops from the uncontrolled production of ROS, which exceeds the body’s antioxidant defense mechanisms [4]. The human body produces ROS through its metabolic operations, with the highest production occurring in the mitochondrial electron transport process [5]. ROS are naturally produced through metabolic processes but when environmental stressors or infections or toxic substances cause an imbalance, the ROS levels rise to dangerous heights and generate major cellular injuries [6,7]. The accumulation of ROS in spermatozoa leads to membrane damage that results in decreased mobility and reduced survival rates [8]. Empirical evidence suggests that ROS levels exceed normal ranges in 30–80% of male infertility cases, which results in DNA, protein, and lipid damage in sperm cells [9]. The negative effects of oxidative changes in sperm cells disrupt capacitation function, which is essential for successful fertilization [10,11]. While the primary focus of this review is on human infertility, relevant animal and in vitro studies were included where they provide essential mechanistic insights into OS and miRNA regulation that cannot yet be fully elucidated in humans.

Research indicates that unbalanced OS in ovarian follicular fluid microenvironments creates adverse effects on oocyte maturation [12,13] and impairs embryonic development [14,15] and leads to negative pregnancy outcomes [16]. DNA damage along with persistent OS affects the regulatory function of apoptosis-related miRNA expression in granulosa cells surrounding oocytes [17]. Several findings report conflicting results regarding polycystic ovary syndrome (PCOS), while multiple studies confirm that women with this condition have higher risks of cardiovascular disease due to obesity, insulin resistance, and hyperandrogenism. The elevated levels of subclinical cardiovascular disease (CVD) markers and increased OS levels heighten the risk for this population [18,19].

OS plays a fundamental role in the pathogenesis of endometriosis. An imbalance of reactive oxygen species (ROS) levels in relation to antioxidant defenses promotes inflammatory responses, cellular damage, and apoptosis in ectopic endometrial tissue [20]. Studies show that endometriosis patients have higher ROS levels and malondialdehyde (MDA) concentrations along with reduced antioxidant defenses, indicating that oxidative stress plays a role in disease progression and symptom development [21]. 

OS contributes to premature ovarian insufficiency (POI) by inducing mitochondrial dysfunction. Excess OS causes damage to mitochondrial DNA while simultaneously decreasing adenosine triphosphate (ATP) production, leading to the deterioration of both oocytes and granulosa cells, and thus reducing ovarian reserve [22,23]. Mitochondria defects and redox imbalances disrupt the process of folliculogenesis, demonstrating their critical function in ovarian health [24].

Therefore, the present review aims to provide a comprehensive and integrative overview of the mechanistic interplay between microRNAs and oxidative stress in human infertility. Specifically, it explores how miRNAs modulate redox balance, mitochondrial function, apoptosis, and steroidogenesis in both male and female reproductive systems. This review differs from previous works by consolidating molecular, cellular, and clinical perspectives to highlight the bidirectional regulation between oxidative stress and miRNAs, emphasizing their potential as diagnostic biomarkers and therapeutic targets. By delineating these interconnections, we aim to advance the understanding of redox-sensitive miRNA networks and their translational implications in reproductive medicine.

## 2. Materials and Methods

This study was designed as a narrative review following a structured but non-systematic approach. From 2015 to 2025, literature searches were conducted in PubMed, Scopus, and Google Scholar using the keywords “microRNAs”, “oxidative stress”, “ROS”, and “infertility”. Several combinations of Boolean operators (AND/OR) were used. Additionally, the “snowball literature searching method” was applied to identify further relevant sources from the reference lists of selected articles. Two reviewers (I.V. and A.P.) separately screened the titles and abstracts, while a third reviewer (T.K.) settled any disputes.

We included peer-reviewed studies addressing miRNA–OS interactions in reproductive tissues or fluids. Studies lacking molecular or mechanistic data, conference abstracts, and non-English research were among the exclusion criteria. The data were qualitatively compiled by study type (human, animal, in vitro), important miRNAs, and mechanistic targets because this was a narrative review, and neither a PRISMA checklist nor a meta-analytic synthesis was conducted. A formal risk of bias and quality assessment was also not performed due to the narrative nature of this review.

## 3. Biogenesis and Function of miRNAs in Reproduction

MiRNAs are small, endogenous, non-coding RNA molecules that consist of approximately 19–25 nucleotides and contribute to post-transcriptional gene regulation [25]. The main pathway of miRNA biogenesis includes the RNA polymerase II/III transcription of primary miRNAs (pri-miRNAs) followed by processing through Drosha-DGCR8 complex in the nucleus and then exportin-5 transporting precursor miRNAs to the cytoplasm where Dicer and its associated proteins achieve final maturation. Mature miRNAs bind to RNA-induced silencing complex (RISC) to regulate gene expression through 3′UTR targeting of mRNAs, although interactions with 5′UTR and coding sequence have also been documented [19].

In reproduction, miRNAs are essential for both male and female fertility. The regulation of spermatogenesis by miRNAs in males affects both germ and Sertoli cell growth and development. The disruption of miRNA biogenesis enzymes DICER and DROSHA leads to extensive spermatogenic failure in animal models, including impaired germ cell development, increased programmed cell death, and infertility [26].

In females, miRNAs are expressed in theca cells, granulosa cells, and are present in follicular fluid, where they regulate essential reproductive mechanisms including folliculogenesis, steroidogenesis, and development [27]. Research on PCOS shows that altered miRNA profiles occur in ovarian tissue, serum, and adipose tissue, reflecting their systemic role in metabolic and endocrine regulation. Importantly, several miRNAs, including miR-21, miR-93, and miR-146a, have consistently been linked to dysregulated insulin signaling and oxidative stress responses in PCOS [28]. However, depending on the biological matrix examined, some studies show contradictory trends. These differences could stem from variations in the stage of follicular growth studied, patient heterogeneity, or sample preparation. Although most of the available data are correlational and based on small cohorts, miRNAs have potential as biomarkers of ovarian function as both functional regulators and circulating molecules [29]. The dual role of miRNAs, to regulate gene expression and function as diagnostic tools, underscores their fundamental role in fertility regulation [30].

## 4. Functions of miRNAs in Reproduction

### 4.1. Male Infertility

OS represents a key pathological mechanism in varicocele-induced male infertility, with miRNAs emerging as essential post-transcriptional regulators of this process. Three OS-associated miRNAs—miR-21, miR-34a, and miR-122a—were found to be substantially decreased in the spermatozoa of men with severe varicocele, coinciding with elevated levels of the lipid peroxidation marker MDA [31]. The survival of spermatogonial stem cells depends on miR-21, whose depletion triggers apoptosis, while miR-34a acts through p53-dependent apoptotic pathways to influence sperm motility and OS resistance. The testis-specific regulator miR-122a also shows reduced expression under high ROS conditions, potentially impairing chromatin remodeling and germ cell development [32]. These changes collectively imply a breakdown of the network of miRNA-mediated antioxidant defense, suggesting that these miRNAs are active regulators of redox homeostasis rather than just indicators. However, the majority of the data currently available are correlational in nature, without consistent quantification across cohorts or functional validation [33]. Future research using multicenter sampling and mechanistic assays is necessary to confirm whether these alterations are compensatory or causative in the pathophysiology of varicocele-related infertility [34]. 

Emerging data implicate miR-145 in the regulation of OS-related sperm dysfunction in varicocele. Infertile men with varicocele present reduced miR-145 expression, while MLH1 expression was elevated, correlating with impaired DNA integrity and diminished antioxidant capacity. The antioxidant levels showed strong positive associations with sperm concentration, motility, and morphology, and an inverse relationship with seminal oxidative damage. These findings suggest that dysregulated miR-145 may contribute to oxidative imbalance and compromised sperm quality, highlighting its potential as a biomarker and mechanistic player in male infertility [35].

The study by Mostafa et al. investigates how seminal miRNAs miR-122 and miR-181a together with miR-34c-5 regulate OS and apoptosis in infertile men who have varicocele [1]. The seminal plasma of oligoasthenoteratozoospermic (OAT) men with varicocele showed reduced levels of miR-122 and miR-181a and miR-34c-5 compared to both healthy fertile men and infertile men without varicocele. The measurements of these miRNAs demonstrated a positive relationship with both sperm parameters and the antioxidant enzyme glutathione peroxidase (GPx) and the anti-apoptotic marker BCL2. The levels of these miRNAs showed a negative association with the pro-apoptotic protein BAX and MDA, which serves as a marker for lipid peroxidation. These miRNAs show promise as non-invasive biomarkers for oxidative damage and reproductive dysfunction in varicocele-related infertility through their regulation of male fertility pathways, including OS and apoptosis [1]. 

Asthenozoospermia is a major cause of male infertility and is closely linked to impaired energy metabolism and OS. Recent research has shown that elevated miR-24-3p levels in asthenozoospermic sperm reduce glycogen synthase kinase 3 beta (GSK3β) expression, leading to disrupted ATP production and weakened antioxidant defenses [36]. A novel therapeutic approach employing Sertoli cell-derived small extracellular vesicles (SC-sEVs) loaded with a miR-24-3p inhibitor successfully traversed the blood–testis barrier and delivered the inhibitor directly into germ cells. In a mouse model, this approach restored GSK3β expression, improved mitochondrial energy metabolism, reduced oxidative damage, and enhanced sperm motility, fertilization success, and litter size [37]. By demonstrating the therapeutic potential of miRNA-based nanodelivery systems, these findings offer strong preclinical evidence that targeted miRNA suppression can restore oxidative stress-mediated sperm abnormalities. However, species variations, a lack of long-term safety evidence, and the difficulty of scaling vesicle-based distribution make translation to clinical application questionable [38]. Finding more precise molecular targets for human trials may be facilitated by combining these insights with more general redox signaling pathways [37].

MiR-34c has emerged as a key regulatory miRNA in spermatogenesis and OS–induced male infertility. Elevated miR-34c expression has been reported in men with moderate oligoasthenoteratozoospermia (OAT), correlating with increased DNA fragmentation and higher MDA levels, a marker of lipid peroxidation. Its expression varies across infertility subtypes—reduced in azoospermia—suggesting that miR-34c participates in a context-dependent regulatory feedback loop rather than a uniform pathological pattern [32,39]. NOTCH family genes have been identified as miR-34c targets, linking this molecule to germ cell differentiation and survival. Importantly, miR-34c interacts closely with p53, a central regulator of DNA damage response and apoptosis. While some studies indicate that miR-34c expression can occur independently of p53, others show that p53 activation drives its upregulation, thereby promoting germ cell apoptosis under OS [40,41]. This dual activity suggests that miR-34c may function in the redox stress network as both an executor and a protector, enhancing apoptosis when oxidative stress surpasses repair capacity and preserving genomic integrity at low stress levels. However, the evidence that is currently available is mostly correlative and derived from small human cohorts [42]. Collectively, these findings highlight the role of miR-34c in maintaining normal spermatogenesis and mediating OS-induced DNA damage and apoptosis, and suggest its potential utility as both a biomarker and a therapeutic target in male infertility [43].

In a study analyzing semen samples, men with reduced sperm motility (<20%) exhibited significantly higher expression of miR-425-3p alongside elevated MDA levels, indicating increased oxidative lipid damage. Importantly, this upregulation of miR-425-3p correlated with poorer semen quality, while lower expression was associated with beneficial microbial profiles, particularly the presence of Bacillus and absence of Neisseria [44]. These findings suggest that miR-425-3p acts as a molecular link between OS and impaired sperm function, providing mechanistic insights into male infertility and pointing to its potential as a therapeutic or diagnostic target [44].

In a study by Huang et al., Porcine Reproductive and Respiratory Syndrome Virus (PRRSV) infection downregulates circ-107191, releasing miR-34c to suppress RAD54L, which promotes apoptosis, OS, and disruption of the blood–testis barrier. These changes impair testosterone production and germ cell maintenance, ultimately compromising male fertility. Importantly, restoring circ-107191 or inhibiting miR-34c reverses these effects by reducing oxidative damage, supporting proliferation, and stabilizing endocrine function. This pathway underscores the broader principle that dysregulated miRNAs can exacerbate OS and reproductive failure [45].

According to Anwar et al., miR-155 was found to play a fundamental role as it is involved in the regulation of inflammatory and oxidative responses in male reproductive system [46]. According to the study, there is a significant upregulation in miR-155 in infertile men with varicocele or inflammatory conditions, and this is positively correlated with pro-inflammatory cytokines such as Tumor Necrosis Factor α (TNF-α). In addition, high miR-155 expression is associated with increased OS and a decrease in sperm quality. This may occur due to the downregulation of anti-inflammatory and antioxidant pathways by miR-155, leading to testicular oxidative damage and spermatogenic impairment. These results indicate that miR-155 is a key molecule in inflammation-induced oxidative stress and a potential biomarker for diagnosing oxidative pathology in male infertility [46].

Another study identified miR-125a-5p as an essential factor in regulating the quality of sperm and the early embryo development in the context of aging. The authors observed that miR-125a-5p expression is significantly increased in the sperm of two aging mouse models and positively correlated with elevated DNA fragmentation index (DFI). Functional analyses revealed that miR-125a-5p impairs mitochondrial function, reduces ATP production, increases intracellular ROS levels, and induces DNA damage in germ cells [47].

In the comparative transcriptomic analysis of spermatozoa with distinct quality profiles, several miRNAs upregulated in the high-quality (S6) group, were identified as regulators of oxidative stress-related pathways. Bioinformatic analysis revealed that miR-125b and miR-532 form a regulatory network centered around E2F1 and E2F3, two transcription factors known to mediate oxidative stress responses and redox imbalance. In addition, the broader group of S6-associated miRNAs was predicted to target genes involved in mechanistic Target of Rapamycin (mTOR) signaling, apoptosis, and senescence. All the last-mentioned processes are linked to ROS dynamics. The observed promoter hypomethylation of these target genes in both S6 and S0 groups further supports the idea that miRNA-mediated post-transcriptional inhibition, rather than transcriptional silencing, is the key regulatory mechanism. This underscores the potential role of sperm-borne miRNAs in modulating OS and maintaining cellular homeostasis within the male reproductive system [48].

### 4.2. Female Infertility

High-throughput sequencing of the porcine granulosa cells under H_2_O_2_-induced OS revealed a distinct profile of 55 differentially expressed miRNAs (DEmiRNAs), including 38 upregulated and 17 downregulated species. Functional annotation of these DEmiRNAs and their predicted mRNA targets indicated significant enrichment in pathways associated with OS regulation, such as Forkhead box O (FoxO), Phosphoinositide 3-kinase/Protein Kinase B (PI3K-Akt), mTOR, and Transforming Growth Factor β (TGF-β) signaling. Notably, ssc-miR-424 and ssc-miR-27b emerged as central regulatory factors within the miRNA–mRNA interaction network, suggesting their critical role in modulating cellular responses to ROS, including proliferation, apoptosis, and hormone secretion. These findings support the involvement of specific miRNA-driven regulatory paths and their role in the maintenance of granulosa cell homeostasis under OS conditions [2].

Recent evidence proves that miR-642a-5p plays a critical role in the regulation of OS and mitochondrial dysfunction and granulosa cell senescence in women with POI. In models of H_2_O_2_-induced injury, there was a significant increase in miR-642a-5p, which downregulates FOXO1, a key factor in the antioxidant defense system. The exosomal circBRCA1, enriched in mesenchymal stem cell-derived exosomes (H-Exs), acts as a competing endogenous RNA (ceRNA) that sponges miR-642a-5p, thereby relieving its inhibitory effect on FOXO1. This molecular interaction restores mitochondrial function and reduces ROS levels, as evidenced by elevated mitochondrial membrane potential, ATP production, and expression of antioxidant enzymes such as Gpx and Superoxide Dismutase 2 (SOD2). Inhibition of miR-642a-5p or overexpression of circular BRCA1 (circBRCA1) significantly attenuates oxidative damage and cellular senescence, indicating the therapeutic potential of the circBRCA1/miR-642a-5p/FOXO1 axis in protecting granulosa cells from OS [49].

Dysregulated miRNAs in PCOS cause ovulatory problems and infertility through their influence on OS as well as metabolic disturbances. The oocyte maturation process and fertility are negatively affected by elevated levels of miR-18b, miR-146a, and miR-135a in PCOS patient follicular fluid because these miRNAs disrupt steroidogenesis by altering progesterone, estradiol, and testosterone production. The presence of miR-93 and miR-21 in PCOS patients correlates with follicular dysfunction and hyperandrogenism, while their association with insulin resistance promotes OS. The nuclear factor kappa-light-chain-enhancer of activated B cells (NF-Κb) pathway becomes regulated by miR-146a as it executes its dual function in inflammation and OS. The PI3K/AKT pathway which regulates insulin signaling and maintains oxidative balance is negatively affected by the elevated miR-133a-3p in obese PCOS patients. The abnormal miRNA expression patterns lead to hormonal imbalances and inflammation and oxidative damage in ovarian tissues, thus indicating their value as both diagnostic markers and therapeutic options for PCOS-related infertility [19].

Evidence from various studies indicates multiple miRNAs regulate OS responses in ovarian dysfunctions including polycystic ovary syndrome (PCOS) and premature ovarian failure (POF) by controlling pathways that affect granulosa cell survival and hormone production. The miR-15b microRNA leads to premature ovarian failure by disrupting autophagy processes while decreasing ROS clearance ability in mouse granulosa cells, which causes oxidative stress and follicle destruction. The miR-379-5p microRNA inhibits DNA repair functions and cell growth of granulosa cells (GCs), which worsens ovarian failure. The PCOS condition develops because miR-335-5p expression decreases, thereby enabling SGK3 overexpression to stimulate abnormal GC proliferation, and miR-135a and miR-9119 increase GC apoptosis, which may occur through stress-sensitive cellular pathways. These miRNAs control oxidative damage responses as well as energy metabolism and cell fate decisions to play a crucial role in the development of infertility-related ovarian disorders through redox-sensitive molecular networks [26].

In PCOS, the AK097578/miR-548i/IDH1 axis exemplifies how microRNAs modulate OS in ovarian cells. The lncRNA AK097578 acts as a sponge for miR-548i, thereby maintaining Isocitrate Dehydrogenase 1 (IDH1) expression, which is essential for regulating the NADP+/NADPH balance and protecting cells from ROS-induced damage. Downregulation of IDH1 disrupts redox homeostasis, leading to increased OS, impaired cell proliferation, and heightened susceptibility to follicular atresia and ovarian aging. This pathway underscores the critical role of miRNA-mediated regulation in ovarian OS and highlights potential therapeutic targets for preserving reproductive function [50].

Te Liu et al. found that delivering miR-146b-5p via Polylactic-co-glycolic acid (PLGA) nanoparticles protected granulosa cells under a high-fat, high-sugar diet by reducing atresia, boosting ATP and Superoxide Dismutase (SOD) activity, and improving cell cycle progression. In mice, miR-146 treatment increased ovarian weight, follicle numbers, and estrogen and progesterone levels while lowering androgens. These effects, linked to suppression of the Dab2ip/Ask1/p38-MAPK pathway and reduced DNA damage, suggest miR-146 as a potential therapy for mitigating OS and preserving ovarian function [51].

The research of Jueraitetibaike et al. demonstrates that miR-425-5p functions as an essential regulator of OS-induced oocyte aging because its levels increase in aging mouse oocytes and after in vitro hydrogen peroxide (H_2_O_2_) treatment. This miRNA becomes activated through ROS accumulation and disrupts oocyte maturation and spindle assembly while increasing meiotic errors. The research findings showed that blocking miR-425-5p resulted in higher oocyte maturation success rates and correct chromosomal alignment, thus indicating the severe effects of this miRNA during OS. The miR-425-5p regulates Crebzf expression, which functions as a critical transcription factor for chromatin remodeling and histone H3K4 trimethylation (H3K4me3) to control oocyte development gene silencing. Overexpression of miR-425-5p suppresses Crebzf expression, which reduces H3K4me3 levels and leads to chromatin structural defects that diminish developmental competence in aged oocytes. The in vivo silencing of miR-425-5p leads to Crebzf expression recovery and preserves spindle structure along with the restoration of H3K4me3-dependent transcriptional gene silencing. These research findings establish miR-425-5p as a redox-sensitive epigenetic regulator with therapeutic potential for treating oxidative stress-induced oocyte decline and age-related fertility reduction [52].

The levels of miR-484 increase substantially in granulosa cells and ovarian tissue when 3-nitropropionic acid creates excessive ROS production through OS. The elevated expression of miR-484 leads to compromised antioxidant mechanisms while simultaneously breaking down mitochondrial processes and inducing apoptosis through Bcl-2-associated X protein (BAX) and cleaved caspase-3 and cytochrome c, while decreasing B-cell lymphoma 2 (BCL2) levels. The miR-484 exerts its effects on cells by directly targeting Sestrin2 (SESN2), which plays a central role in maintaining mitochondrial equilibrium and activating antioxidant signaling through the AMPK–FOXO1–SIRT1 pathway. LINC00958 functions as an endogenous RNA that counteracts the negative effects of miR-484. In vivo experiments showed that blocking miR-484 expression helped restore ovarian function while protecting against oxidative damage, which demonstrates its potential as a therapeutic target for ovarian dysfunction caused by OS [53].

The inflammatory signaling pathways and OS mechanisms in endometriosis are primarily controlled by miRNAs. Several miRNAs including miR-199a, miR-16, miR-138, and miR-182 are downregulated in ectopic endometrial stromal cells, leading to the activation of the NF-κB pathway and ROS production. The IκB kinase (IKK) expression is suppressed by miR-199a and miR-16, which blocks NF-κB nuclear translocation and subsequent cytokine production. The RELA (p65) target of miR-182 leads to elevated Cyclooxygenase-2 (COX-2) expression and inflammatory signaling. The NF-κB pathway receives additional input from miR-20a through Dual-Specificity Phosphatase 2 (DUSP-2) suppression and Extracellular signal-Regulated Kinase/Hypoxia-Inducible Factor 1α (ERK/HIF-1α) activation, while miR-215-5p regulates C-X-C Motif Chemokine Ligand 2 (CXCL2) to affect neutrophil recruitment. The inflammatory microenvironment is shaped by additional miRNAs such as let-7b and miR-301a-3p, which influence the polarization of immune cells. These findings indicate that endometriosis-related OS emerges from immune and inflammatory pathway dysregulation through miRNA expression alterations that could serve as therapeutic targets [54].

The study by Khan et al. identified a strong association between OS and the changes in the expression of certain miRNAs in follicular fluid, especially with regards to intrafollicular melatonin levels. Among the analyzed miRNAs, miR-132-3p was significantly upregulated in patients with higher melatonin levels, who also exhibited lower ROS, Thiobarbituric Acid Reactive Substances (TBARS), and 8-hydroxy-2′-deoxyguanosine (8-OHdG) levels, as well as enhanced total antioxidant capacity (TAC). On the other hand, in patients with low melatonin (≤30 pg/mL), miR-132-3p levels were significantly decreased, a state that was associated with increased OS and poor IVF outcomes, including lower embryo quality. Moreover, miR-132-3p expression was positively associated with high-quality day 3 embryos, suggesting its potential role in promoting an oxidative balance conducive to oocyte maturation. Bioinformatic analyses indicated that miR-132-3p may be involved in key developmental and redox-regulating pathways, including the PI3K-Akt, TGF-β, and Hippo signaling pathways. These findings highlight the antioxidant-modulating function of miR-132-3p in the follicular microenvironment and its probable utility as a non-invasive biomarker of oocyte developmental potential and oxidative status during assisted reproduction [17].

The let-7 microRNA family shows increased expression in the follicular fluid (FF) and GCs of infertile patients with ovarian endometriosis (OEM) while impairing folliculogenesis. The most notable increase in the let-7 family members let-7a-5p, let-7c-5p, and let-7d-5p was associated with elevated progesterone levels and a disrupted progesterone to estrogen ratio. GCs exposed to let-7 showed reduced proliferation rates and disrupted steroidogenesis along with higher ROS production and decreased ATP production. Let-7 functions as a direct regulator of Insulin-like Growth Factor 1 Receptor (IGF1R). The reduction in IGF1R expression caused by let-7 leads to impaired GC metabolic function and diminished antioxidant capabilities. The inhibition of let-7 or IGF-1/FSH supplementation helps to reverse the OS and proliferative deficits that occur during these experiments. Research shows that the let-7/IGF1R axis functions as an essential mechanism to maintain redox homeostasis and ovarian function in OEM and suggests therapeutic opportunities to treat oxidative stress-related infertility [55].

Table 1 summarizes the key miRNAs that regulate OS across different infertility-related conditions. The table presents information about the miRNAs mentioned in the results along with their molecular functions and their clinical relevance in the OS–infertility interaction. To enhance transparency, Table 2 summarizes all human clinical studies investigating miRNA–OS interactions in infertility, outlining their design, biological samples, analytical methods, and principal findings.

## 5. Discussion

**Figure 1:**
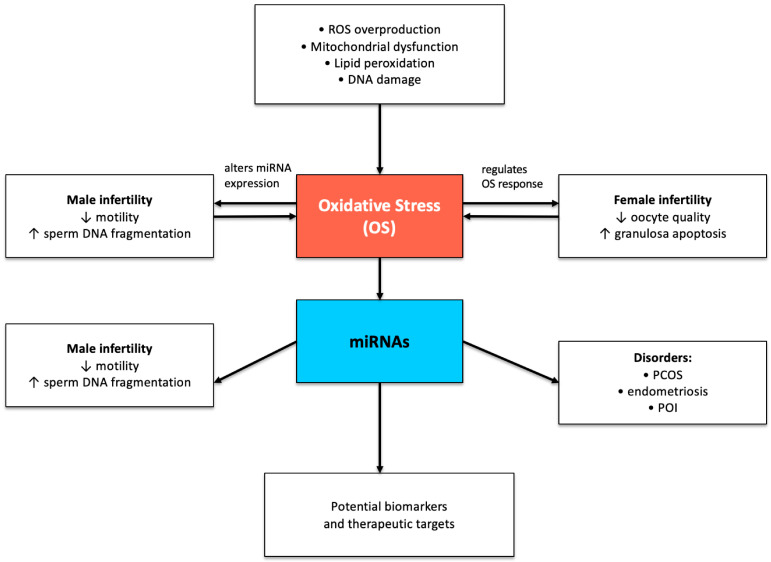
Schematic representation of the bidirectional relationship between OS and miRNAs (miRNAs) in human infertility. OS, characterized by excessive ROS production, mitochondrial dysfunction, lipid peroxidation, and DNA damage, alters the expression of multiple miRNAs. In turn, specific miRNAs modulate OS responses by regulating antioxidant defense, mitochondrial integrity, and apoptosis. This reciprocal interaction contributes to impaired sperm function, oocyte quality, and granulosa cell viability, and is implicated in infertility-related disorders such as PCOS, endometriosis, and POI. miRNAs also hold promise as non-invasive biomarkers and potential therapeutic targets in OS-associated reproductive dysfunction. ↓ means decreased and ↑ means increased.

The present review examines how OS-regulating miRNAs influence male and female infertility through their effects on redox homeostasis, cellular metabolism, apoptosis, steroidogenesis, and gametogenesis. This study reveals that various miRNAs become dysregulated in polycystic ovary syndrome (PCOS), premature ovarian insufficiency (POI), endometriosis (OEM), varicocele and age-related reproductive decline, thus indicating their fundamental role in reproductive dysfunction caused by oxidative stress. Figure 1 presents a schematic representation of the bidirectional relationship between OS and miRNAs in human infertility. Certain studies underscore the crucial role of miRNAs such as miR-146a and miR-155 in various conditions. MiR-146 was found to protect against OS-induced ovarian dysfunction [57] and miR-155 is a potential marker of subfertility in men [57].

The female reproductive disorders PCOS, POI, and endometriosis present dysregulated miRNAs that lead to OS and hormonal imbalance and granulosa cell dysfunction. The miRNAs miR-146a, miR-135a, and miR-21 disrupt the NF-κB, PI3K/Akt, and FOXO1 pathways, which results in impaired steroidogenesis and affects oocyte maturation and fertility. The let-7 family upregulation in ovarian endometriosis disrupts GC metabolism and redox balance through IGF1R inhibition, which supports the role of redox-sensitive miRNAs in female infertility. Additionally, the let-7 family has been associated with receptivity modulation, as it was found in various compartments of the endometrium [58].

The expression of miRNAs including miR-155, miR-34a, miR-122a, and miR-125a-5p changes in males with varicocele and aging-related OS. These miRNAs regulate essential cellular processes including apoptosis, chromatin integrity, and mitochondrial function. Another study conducted on a rat-model concluded that testicular damage caused by varicocele is associated with OS, DNA damage, and increased miR-34 expression along with suppression of SIRT1/FOXO axis [59]. MiR-155 activates inflammation through NF-κB but miR-125a-5p and miR-425-5p disrupt energy production and meiotic stability, which demonstrates how miRNA-mediated oxidative imbalance affects sperm quality and male fertility.

Multiple miRNAs across different conditions share common pathways related to OS. The regulatory axes that control cellular processes include FOXO1, which is targeted by miR-642a-5p [44,49] and by miR-221-3p, thereby regulating apoptosis of ovarian GC in older women with Diminished Ovarian Reserve (DOR) [60]. The TGF and TGF-β pathways are associated with ssc-miR-424, ssc-miR-27b, and miR-132-3p. The research findings demonstrate that TGF-β signaling acts as a primary mechanism for endometrial fibrosis, which leads to intrauterine adhesion (IUA). The TGF-β1-induced fibrotic changes in endometrial stromal cells are counteracted by Immunity and Matrix Regulatory Cells (IMRCs) through suppression of the TGF-β/Smad signaling pathway. The antifibrotic effect occurs through exosomal miRNAs that use paracrine mechanisms to block TGF-β-induced EMT and inflammation. The research indicates that miRNA-based interventions targeting TGF-β signaling pathways could serve as an effective therapeutic approach [61]. Additionally, miR-132-3p has been proved as the most predictive for fertilization rate [62].

Reproductive competence in both sexes depends on the coordination of vital cellular processes like metabolism, survival, and immunological signaling, which are all regulated by the pathways involving OS-responsive miRNAs. The potential of these redox-sensitive reproductive networks as non-invasive indicators and therapeutic targets is highlighted by the frequent identification of specific miRNAs within them [63]. For instance, follicular fluid expression of miR-132-3p is correlated with oxidative balance, melatonin levels, and embryo quality, indicating that it may improve the predictive precision of IVF results [17]. Likewise, it has been demonstrated that experimentally inhibiting harmful miRNAs like miR-484 or miR-425-5p restores mitochondrial function and cellular homeostasis, offering encouraging mechanistic insights into potential therapeutic approaches [52,53].

However, there are several restrictions on this evaluation. A direct comparison is more challenging due to the heterogeneity of the available research, which includes a variety of experimental models, sample types, and analytical methodologies [64]. In vitro or animal research still provides the majority of the evidence, and only a small number of extensive human studies have confirmed that particular miRNAs are biomarkers of oxidative infertility. The observed relationships may also be impacted by variations in normalization techniques and the possibility of publication bias [34,65].

The field offers substantial promise despite these obstacles. Future studies might focus on creating multicenter human populations, standardizing miRNA quantification procedures, and creating longitudinal studies that relate redox-sensitive miRNA profiles to reproductive outcomes [66]. To verify causal roles, functional in vivo studies employing miRNA mimics or inhibitors are also crucial. Furthermore, the combination of multi-omics data and AI-based modeling could enhance our understanding of the networks of interactions between miRNA and ROS and hasten the conversion of these discoveries into treatments and diagnostics for infertility brought on by OS [67,68,69].

## 6. Conclusions

The interplay between OS and miRNAs functions as a vital mechanism for regulating reproductive processes in both genders. The growing evidence demonstrates that miRNAs function both as controllers and responders to redox imbalances, affecting sperm development as well as egg maturation and embryonic development. Research has identified miR-21 and miR-34a and miR-155 and miR-132-3p as promising biomarkers for therapeutic interventions in varicocele, PCOS, endometriosis, and POI. Despite the notable progress in the field, important gaps still exist regarding the temporal dynamics as well as tissue-specific functions of redox-sensitive miRNAs. Research in the future must concentrate on extended longitudinal studies and clinical validation methods to develop diagnostic and therapeutic applications of miRNAs in OS-related infertility.

## Figures and Tables

**Table 1:** OS-related miRNAs implicated in infertility.

miRNA	Function/Action	Condition	Experimental Model	Clinical Relevance	Reference
miR-34c, miR-21, miR-122a	Anti-oxidative, anti-apoptotic	Varicocele	Human semen samples (in vivo; infertile men with varicocele)	↓ in OAT; correlate with ROS and apoptotic markers; potential non-invasive fertility biomarkers	[1]
ssc-miR-424, ssc-miR-27b	Regulate ROS, apoptosis, hormone secretion	OS	Porcine granulosa cells (in vitro; hydrogen peroxide-induced OS)	Maintain GC homeostasis via FoxO, PI3K-Akt, mTOR, TGF-β pathways	[2]
miR-132-3p	Antioxidant regulation, promotes embryo quality	Follicular OS, IVF	Human follicular fluid (ex vivo; patients with unexplained infertility)	↑ with melatonin; biomarker of oocyte quality (PI3K-Akt, TGF-β, Hippo pathways)	[17]
miR-18b, miR-146a, miR-135a	Disrupt steroidogenesis	PCOS	Human (ex vivo; granulosa cells and follicular fluid from PCOS patients)	Affect oocyte maturation and fertility	[19]
miR-93, miR21	Follicular dysfunction, ↑ androgens, and insulin resistance	PCOS	Human serum and ovarian tissue (ex vivo; PCOS patients vs. controls)	Linked to OS and hyperandrogenism	[19]
miR-146a	Regulates NF-κΒ signaling	PCOS	Human granulosa cells (ex vivo; PCOS patients)	Modulates inflammation and OS modulation	[19]
miR-133a-3p	Inhibits PI3K/AKT, worsens insulin signaling	Obese PCOS	Human adipose tissue and ovarian (ex vivo; obese PCOS women)	Promotes OS and insulin resistance; potential therapeutic target	[19]
miR-15b	Impairs autophagy, ↓ ROS clearance	POF	Mouse (in vitro; granulosa cells, OS model)	Leads to OS and follicle destruction	[30]
miR-379-5p	Inhibits DNA repair, GC proliferation	POF	Mouse (in vitro; granulosa cells, OS model)	Promotes GC dysfunction and ovarian failure	[30]
miR-335-5p	↓ expression leads to SGK3 overexpression	PCOS	Human (ex vivo; ovarian tissue from PCOS patients)	Causes abnormal CG proliferation	[30]
miR-135a	Promotes GC apoptosis via stress pathways	PCOS	Human (ex vivo; granulosa cells from PCOS patients)	Contributes to follicular damage and OS	[30]
miR-21, miR-34a, miR-122a	Anti-apoptotic (SSC survival); motility and chromatin regulation	Varicocele	Human (in vivo; semen samples from infertile men with grade III varicocele and fertile controls)	↓ expression linked to ROS and apoptosis; biomarkers of oxidative damage	[34]
miR-145	Regulates OS, DNA integrity, antioxidant capacity	Varicocele	Human (in vivo; semen samples from infertile men with grade III varicocele and fertile controls)	↓ linked to poor sperm quality and oxidative imbalance	[35]
miR-24-3p	Targets GSK3β leads to ↓ ATP and antioxidant defense	Asthenozoospermia	Mouse (in vivo; gossypol-induced asthenozoospermia model, treated with SC-sEV@miR-24-3p inhibitor)	Inhibition restores motility, metabolism, and fertility; therapeutic target	[37]
miR-34c	Regulates spermatogenesis; OS-induced apoptosis	OAT, varicocele, asthenozoospermia	Human (in vivo; semen samples from infertile men with MOAT, SOAT, OA, and NOA compared to normozoospermic)	Correlates with DNA damage and ROS; biomarker and therapeutic target	[1]
miR-24-3p	Targets GSK3β leads to ↓ ATP and antioxidant defense; regulates sperm energy metabolism and ROS	Asthenozoospermia	Mouse (in vivo; gossypol-induced asthenozoospermia model treated with SC-sEV@miR-24-3p-inhibitor)	↑ expression linked to poor sperm motility and OS; inhibition restores motility, metabolism, fertilization, and litter size; potential therapeutic target	[37]
miR-34c	Regulates spermatogenesis; linked to apoptosis and oxidative stress	Oligoasthenoteratozoospermia (MOAT, SOAT), NOA	Human (in vivo; semen samples from infertile men grouped by semen analysis)	Semen samples from infertile men grouped by semen analysis) ↑ expression in infertile groups associated with higher DNA fragmentation and oxidative stress; potential biomarker for male infertility	[43]
miR-425-3p	Associated with OS and semen quality; regulates sperm motility	Asthenozoospermia/low sperm motility	Human (in vivo; semen samples from men with sperm motility < 20% vs. >20%)	↑ expression correlated with higher oxidative stress (MDA) and reduced sperm motility; potential biomarker for impaired semen quality	[44]
miR-34c	Promotes apoptosis, oxidative stress, and germ cell dysfunction via RAD54L suppression	PRRSV infection-induced testicular dysfunction	Porcine testicular cells (ex vivo)	Elevated miR-34c disrupts blood–testis barrier, increases inflammation and apoptosis, reduces testosterone; inhibition mitigates damage and restores function	[45]
miR-155	Upregulated in testicular tissue under DOX-induced oxidative stress; contributes to apoptosis and inflammation	Doxorubicin-induced testicular injury	Rat (in vivo; Wistar rats treated with DOX ± ACA or B. cereus protease)	Modulation via ACA or B. cereus protease reduces oxidative stress, apoptosis, and restores testicular function	[46]
miR-34c	Germ cell-specific; aberrantly expressed under DOX stress; linked to impaired spermatogenesis and apoptosis	Doxorubicin-induced testicular injury	Rat (in vivo; Wistar rats treated with DOX ± ACA or B. cereus protease)	Protective interventions normalize miR-34c expression, improve spermatogenesis and testicular histology	[46]
miR-125a-5p	Upregulated in aging sperm; suppresses mitochondrial function, increases DNA damage, perturbs embryo development	Aging males (advanced paternal age)	Human sperm; functional validation in GC2 mouse spermatocyte cell line	Elevated miR-125a-5p is linked to poor sperm DNA integrity and early embryo developmental delay; potential biomarker or therapeutic target for paternal age-related infertility	[47]
miR-34, miR-30, miR-122, miR-20, miR-182, miR-191	Upregulated in good-quality sperm; regulate cell death, survival, OS, and metabolism	Ovarian dysfunction (HFHS diet)	Human (ex vivo; semen samples)	Potential biomarker for sperm quality	[48]
miR-642a-5p	FOXO1 (sponged by circBRCA1)	Inhibition alleviates oxidative stress, mitochondrial dysfunction, apoptosis, and senescence; protects ovarian function	Human granulosa cells, rat POI model	Premature ovarian insufficiency (POI); potential therapeutic target to preserve ovarian function	[49]
miR-484	Targets SESN2 leads to ↓ AMPK-FOXO1-SIRT1 signaling	Ovarian OS	Human granulosa cells (in vitro) and mouse models (in vivo)	↑ ROS and apoptosis; blocking miR-484 restores mitochondrial function	[50]
miR-199a, miR-16, miR-182, miR-20a, miR-215-5p, let-7b, miR-301a-3p	Regulate NF-κB, ERK/HIF-1α, CXCL2, immune polarization	Endometriosis	Mouse (in vitro; granulosa cells)	Dysregulation leads to ↑ ROS and inflammation; pro-inflammatory microenvironment	[51]
let-7a-5p, miR-16, miR-182, miR-20a, miR-215-5p, let-7b, miR-301a-3p	Target IGF1R leads to ↓ GC proliferation, ↑ ROS	Ovarian endometriosis (OEM)	Mouse (in vivo; oocytes from reproductively aged females and in vitro oocyte culture)	Impaired steroidogenesis and redox balance; therapeutic let-7/IGF1R axis	[52]

↓ means decreased and ↑ means increased.

**Table 2:** Summary of human clinical studies investigating microRNA–OS interactions in infertility.

Authors	Population/Condition	Sample Type	miRNAs	Assay and Normalization	Key Findings
Mostafa et al., 2016 [1]	Infertile men with varicocele (OAT) vs. fertile controls	Seminal plasma	miR-122, miR-181a, miR-34c-5p	RT-qPCR; U6 snRNA	↓ in infertile men; correlated with GPx and BCL2, inversely with BAX and MDA (oxidative/apoptotic markers).
Ashrafzade et al., 2020 [34]	Varicocele patients (grade III)	Spermatozoa	miR-21, miR-34a, miR-122a	RT-qPCR	Reduced expression correlates with ROS overproduction; potential OS biomarkers.
Hekim et al., 2024 [35]	Varicocele-related infertility	Sperm	miR-145	RT-qPCR; RNU48	↓ miR-145 leads to increased MLH1 and DNA fragmentation; OS biomarker.
Khan et al., 2021 [17]	IVF patients	Follicular fluid	miR-132-3p	RT-qPCR; RNU44	↑ miR-132-3p linked to higher melatonin and embryo quality; ↓ in OS.
Shi et al., 2023 [24]	Women with ovarian endometriosis	Follicular fluid/GCs	let-7a-5p, let-7c-5p, let-7d-5p	qPCR; miR-16	Overexpression leads to ↓ IGF1R, ↑ ROS, impaired folliculogenesis.
Batiha et al., 2025 [44]	Men with reduced sperm motility	Semen	miR-425-3p	RT-qPCR	↑ miR-425-3p associated with MDA levels and poor sperm quality.
Eckersten et al., 2017 [56]	Men with chronic kidney disease (subfertility)	Serum	miR-155	RT-qPCR	↑ miR-155 linked with subfertility and inflammation; possible biomarker of OS-related infertility.

↓ means decreased and ↑ means increased.

## Data Availability

No new data were created or analyzed in this study. Data sharing is not applicable to this article.

## Abbreviations

The following abbreviations are used in this manuscript:

OS	oxidative stress
miRNAs	microRNAs
ROS	reactive oxygen species
PCOS	polycystic ovary syndrome
CVD	cardiovascular disease
MDA	malondialdehyde
POI	premature ovarian insufficiency
ATP	adenosine triphosphate
RISC	RNA-induced silencing complex
OAT	oligoasthenoteratozoospermic
GPx	glutathione peroxidase
TNF-α	Tumor Necrosis Factor α
DFI	DNA fragmentation index
mTOR	mechanistic Target of Rapamycin
DEmiRNAs	differentially expressed miRNAs
TGF-β	Transforming Growth Factor β
FoxO	Forkhead box O
PI3K-Akt	Phosphoinositide 3-kinase/Protein Kinase B
ceRNA	competing endogenous RNA
SOD2	Superoxide Dismutase 2
circBRCA1	circular BRCA1
NF-Κb	nuclear factor kappa-light-chain-enhancer of activated B cells
POF	premature ovarian failure
H_2_O_2_	hydrogen peroxide
H3K4me3	histone H3K4 trimethylation
BAX	Bcl-2-associated X protein
BCL2	B-cell lymphoma 2
SESN2	Sestrin2
IKK	IκB kinase
COX-2	Cyclooxygenase-2
DUSP-2	Dual-Specificity Phosphatase 2
ERK/HIF-1α	Extracellular signal-Regulated Kinase/Hypoxia-Inducible Factor 1α
CXCL2	C-X-C Motif Chemokine Ligand 2
TBARS	Thiobarbituric Acid Reactive Substances
8-OHdG	8-hydroxy-2′-deoxyguanosine
TAC	total antioxidant capacity
FF	follicular fluid
GCs	granulosa cells
OEM	ovarian endometriosis
IGF1R	Insulin-like Growth Factor 1 Receptor
SIRT1	Sirtuin 1
DOR	Diminished Ovarian Reserve
IUA	intrauterine adhesion
IMRCs	Immunity and Matrix Regulatory Cells
PRRSV	Porcine Reproductive and Respiratory Syndrome Virus
PGLA	Polylactic-co-glycolic acid
SOD	Superoxide Dismutase
IDH1	Isocitrate Dehydrogenase 1
GSK3β	glycogen synthase kinase 3 beta
